# Carnitine deficiency in epileptic children treated with a diversity of anti-epileptic regimens

**DOI:** 10.1186/s41983-018-0033-z

**Published:** 2018-11-21

**Authors:** Sherine El Mously, Hadeer Abdel Ghaffar, Remon Magdy, Somaia Hamza, Mohamed Mansour

**Affiliations:** Faculty of Medicine, Fayoum University, Fayoum City, 63611 Egypt

**Keywords:** Carnitine, Epilepsy, AEDs

## Abstract

**Background:**

Carnitine deficiency is relatively common in epileptic patients. The risk factors reported include the combination of valproic acid with other antiepileptic drugs (AEDs), young age, multiple neurologic disabilities, non-ambulatory status, and being underweight.

**Objectives:**

To study the level of carnitine deficiency and its associated risk factors among a group of children with idiopathic epilepsy treated with different AEDs.

**Patients and methods:**

Fifty children with idiopathic epilepsy and 40 age-matched controls were enrolled. For all, serum carnitine level was measured by enzyme-linked immune sorbent assay (ELISA).

**Results:**

The mean carnitine level was lower in cases compared to controls (*p* = 0.04). Patients receiving monotherapy treatment had a high percentage of carnitine deficiency compared to controls (*p* = 0.04). Patients receiving valproate with other AEDs had a lower level of carnitine compared to controls (*p* = 0.03). The age of the patients, the duration of treatment, and the doses of different AEDs were not risk factors for carnitine deficiency.

**Conclusions:**

Carnitine deficiency is common in our population, and the use of valproate with other AEDs is considered the most important risk factor for it in epileptic children.

## Introduction

Carnitine plays essential roles in metabolism specifically the transport of long-chain fatty acids from the cytosol to the mitochondria. The majority of carnitine is obtained from diet, mainly animal products, and only a small fraction is obtained endogenously [1]. Carnitine exists in the body in two forms; L-carnitine or Acetyl-L-Carnitine [2]. The synthesis of these molecules occurs in the kidneys, liver, and brain. Cardiac and skeletal muscles that harbor the highest concentrations of carnitine are not able to synthesize it; and consequently, must acquire it from plasma [3].

Carnitine deficiency may occur secondary to pharmacological therapy such as valproic acid, some inherited metabolic disorders, poor diet or malabsorption of carnitine, increased renal tubular loss, hemodialysis, or peritoneal dialysis [4]. Severe carnitine deficiency can exist due to lack of dietary intake resulting in symptoms similar to dementia [5], muscle weakness, and hepatic dysfunction [6] as well as cardiomyopathy [7].

Risk factors associated with carnitine deficiency include young age, treatment with multiple antiepileptic drugs (AEDs), the presence of multiple neurologic disabilities, non-ambulatory status, and being underweight [8]. Few studies have examined the correlation between each risk factor and carnitine deficiency in children with epilepsy. Our aim in this study is to investigate the level of carnitine deficiency and its associated risk factors among a group of children with idiopathic epilepsy receiving different regimens of AEDs either mono- or polytherapy.

## Patients and methods

This descriptive cross-sectional case control study was conducted in the neuropediatric clinic of Fayoum University Hospitals in the period from January to March 2017. Fifty consecutive children over 6 months old were recruited. The diagnosis of idiopathic epilepsy was applied according to the International League Against Epilepsy 2010 [9]. We included patients with normal motor and mental development, absence of any neurological deficits, and normal brain magnetic resonance imaging (MRI).

The patients were receiving either single or multiple AEDs for 6 months or more. We excluded patients with previous recurrent attacks of encephalopathy and disturbed conscious level, metabolic acidosis, or regression of previous acquired skills, cerebral palsy, and liver or kidney diseases. Also, patients receiving carnitine supplement for 6 months before sampling, those receiving drugs other than AEDs and those under ketogenic diet, were all excluded.

Forty age- and sex-matched children were enrolled in our study as a control group. Those children came to the general pediatric outpatient clinic suffering from tonsillitis or pharyngitis. We insisted for the control group on several points such as no history of seizures or any neurological diseases, and we also excluded cases with gastroenteritis or malnutrition.

All the patients were subjected to full history taking focusing on the type of seizures and its frequency, the age of onset, the doses, and the duration of treatment of AEDs; and family history of epilepsy. This was followed by general and neurological examination considering the symptoms and signs of carnitine deficiency such as muscle weakness, hypotonia, nausea and vomiting, fatigue, recurrent infection, failure to thrive, anorexia, poor concentration, apathy, and headache. All patients were subjected to digital encephalogram (EEG) that was carried either under standardized conditions or after sedative pre-medication as chloral hydrate in non-cooperative children. The EEG tracing was analyzed as regards background activity and presence of generalized or focal epileptogenic discharges.

Total serum carnitine level was measured for all our patients and for the control group. Total human carnitine level was quantitated by enzyme-linked immune sorbent assay (ELISA) based on the biotin double antibody sandwich technology, using commercially available kit (BT Laboratory, Shanghai, China). The whole procedure was performed according to manufacturer guidelines. Serum samples were allowed to clot for 10–20 min at room temperature. Samples were centrifuged at 2000–3000 rpm for 20 min, and supernatants were collected carefully. Then, 40 μl of each sample was added to corresponding well, followed by addition of 10 μl total carnitine (TC) antibodies and 50 μl streptavidin-horseradish peroxidase (HRP). The micro-well plate was then covered with seal plate membrane, shacked gently to mix contents up, and finally incubated at 37 °C for 60 min. For standard wells, 50 μl standard was added. After incubation, contents of the wells were drained and wells were filled with wash solution, plate was kept standing for 30 s, then liquid was drained. Wash was repeated five times.

For final color development, 50 μl chromogen solution A was firstly added to each well followed by 50 μl of chromogen solution B, shacked gently to mix, and finally incubated for 10 min at 37 °C in dark. Finally, 50 μl stop solution was dispensed to each well to stop the reaction. The absorbance (OD) was measured at 450 nm wave length within 10 min after having added the stop solution. Final concentration was then calculated by the linear regression equation of the standard curve. We referred to Zammit V [10] for the range of carnitine level according to the age as shown in Table 1.

**Table 1:** Range of total carnitine levels according to the age

Age	Total carnitine level
First day	23 to 68 nmol/ml
2 to 7 days	17 to 41 nmol/ml
8 to 31 days	19 to 59 nmol/ml
32 days to 12 months	38 to 68 nmol/ml
13 months to 6 years	35 to 84 nmol/ml
7 to 10 years	28 to 83 nmol/ml
11 to 17 years	34–77 nmol/ml

This study was approved by the Faculty of Medicine Research Ethical Committee of Fayoum University. A written consent was taken from the parents of all children participating after being informed about the objectives of the study. The confidentiality of their information was respected, and their right not to participate in the study was ensured.

### Statistical analysis

Data were collected and coded into Microsoft Access, and data analysis was performed using SPSS software version 18 under windows 7. Simple descriptive analysis in the form of numbers and percentages was used for qualitative data, while arithmetic means as central tendency measurement and standard deviations were used as measures of dispersion for quantitative parametric data. For quantitative parametric data, we used the Student’s *t* test to compare measures of two independent groups. Also, one-way analysis of variance (ANOVA) test was used in comparing more than two independent groups. For quantitative non-parametric data, Kruskal-Wallis test was used in comparing more than two independent groups and Mann-Whitney test in comparing two independent groups. Finally, for qualitative data, chi-square test was used to compare two of more than two qualitative groups and bivariate Pearson correlation test to test association between variables. The level *p* ≤ 0.05 was considered the cut-off value for significance.

## Results

Our study included 50 children with idiopathic epilepsy and 40 controls who are age- and sex-matched. Demographic data are shown in Table 2.

**Table 2:** Demographic data of patients and controls

	Cases (*n* = 50)		Controls (*n* = 40)		*p*
	No.	%	No.	%	
Male	23	46%	24	60%	0.2
Female	27	54%	16	40%	
Age 6–24 months	2	4%	6	15%	0.1
Age > 24 months	48	96%	34	85%	
Mean of age ± SD	8.7 ± 3.1		7.4 ± 3.5		0.06

The history taking revealed that 33/50 patients (66%) had generalized tonic clonic seizures, 15/50 (30%) had absence seizures while only 2/50 (4%) had focal fits. Regarding the EEG findings, 26/50 (56%) showed normal EEG while 24/50 (48%) exhibited EEG abnormalities. Focal epileptogenic activity was detected in 10/24 (41.66%), paroxysms of slow spikes and waves (3 Hz) in 10/24 (41.66%), and generalized epileptogenic activity in 4/24 (16.66%). The mean age of first attack among cases was 4.7 ± 3 years old, ranging between 8 months and 11 years old, and mean duration of treatment was 4.2 ± 3.1 years ranging between 6 months and 11 years.

The mean carnitine level was statistically lower in cases compared to controls (*p* = 0.04). There was a high percentage of low carnitine level among cases (*p* = 0.03) as shown in Table 3. All children included with low carnitine level were asymptomatic. There was no statistically significant difference in carnitine level between different age groups (*p* > 0.05) as mentioned in Table 2.

**Table 3:** Carnitine level in different study groups (upper part of the table) and proportion of patients with normal and low carnitine level (lower part of the table)

	Carnitine level				*p*
	Mean		SD		
Cases (*n* = 50)	38.1 nmol/ml		± 29.4		0.04
Control (*n* = 40)	52.2 nmol/ml		± 36.2		
Carnitine level	Cases (*n* = 50)		Control (*n* = 40)		*p*
	No.	%	No.	%	
Normal	18	36%	24	60%	0.03
Low	32	64%	16	40%	

Nineteen patients were receiving treatment from 6 months to 2 years with a mean of carnitine level 40.7 nmol/ml ± 30.6, only 9 patients were receiving treatment for 2–4 years with a mean carnitine level 34.9 nmol/ml ± 34.5 and lastly those who were receiving treatment for a longer duration (> 4 years) were 22 with a mean of carnitine level 37 nmol/ml ± 27.3. There was no statistically significant difference between the three groups regarding the carnitine level (*p* > 0.05).

Our results showed that the group of patients on monotherapy had a statistically significant higher level of carnitine level compared to control group (*p* = 0.04), but no statistical difference in patients treated with polytherapy compared to controls (*p* = 0.07) as shown Table 4.

**Table 4:** Frequency of patients on mono and polytherapy having normal and low carnitine level compared to controls

Treatment	Carnitine level				*p*
	Normal		Low		
	No.	%	No.	%	
Controls	24	60%	16	40%	0.04*
Cases on monotherapy	10	34.6%	19	65.5%	
Controls	24	60%	16	40%	0.07
Cases on polytherapy	8	38.1%	13	61.9%	

A higher percentage of patients with low level of carnitine was found among the group taking valproate either as monotherapy or with other AEDs compared to control group (*p* = 0,05; *p* = 0.03 respectively) (Table 5). Patients receiving valproate and having low carnitine level were 20/28 (71.4%); they were statistically higher in percentage compared to the controls (16/40, 40%) (*p* = 0.01). There was no statistical difference between patients receiving valproate and have normal carnitine level (8/28, 28.6%) and controls (24/40, 60%) (*p* > 0.05). There was no statistical significant difference in carnitine level between patients receiving valproate of less than 20 mg/kg/day and those receiving valproate of more or equal to 20 mg/kg/day (*p* > 0.05). Finally, there was no statistically significant difference among groups of patients receiving valproate and having either low or normal carnitine level regarding the duration of treatment (*p* > 0.05).

**Table 5:** Frequency of patients having normal and low carnitine level among different AEDs groups compared to controls

Variables	Carnitine level				*p*
	Normal		Low		
	No	%	No	%	
Valproate	5	31.2	11	68.7	0.05*
Controls	24	60	16	40	
Valproate + other AEDs	3	25%	9	75%	0.03*
Controls	24	60%	16	40%	
Carbamazepine	3	33.3%	6	66.7%	0.1
Controls	24	60%	16	40%	
Carbamazepine + other AEDs	2	50%	2	50%	0.7
Controls	24	60%	16	40%	
Other AEDs	5	55.6%	4	44.4%	0.8
Controls	24	60%	16	40%	

Other AEDs are phenytoin, levetiracetam, oxi-carbazepine, lamotrigen, and ethosuximide

Moving to the carbamazepine, we had in our study nine patients receiving carbamazepine as monotherapy and another four patients receiving it with other AEDs excluding the valproate. There was no statistically significant difference in carnitine level groups (normal or low) between the patients receiving carbamazepine either as mono- or polytherapy compared to controls (*p* > 0.05) (Table 5). Also, there was no statistical significant difference in carnitine level groups between patients receiving carbamazepine less or more than 15 mg/kg/day (*p* > 0.05). Finally, there was no statistically significant difference in carnitine level groups regarding the duration of treatment with carbamazepine as mono- or polytherapy (*p* > 0.05).

Regarding the patients receiving other AEDs, there was no statistically significant difference in carnitine level among patients receiving these AEDs compared to controls (*p* > 0.05) (Table 5). Also, there was no statistically significant difference in carnitine level in patients receiving other AEDs regarding the duration of treatment (*p* > 0.05).

## Discussion

Carnitine plays a vital role in energy production and fatty acid metabolism. Secondary carnitine deficiency is less severe regarding its clinical impact and much more common than primary carnitine deficiency. It may occur due to or in association with other disorders such as liver and kidney diseases, defects in fatty acid metabolism, or administration of pharmacological agents such as valproic acid [4].

Little data are available on the incidence of carnitine deficiency in epileptic children and the impact of different AEDs on carnitine level. Carnitine deficiency in epileptic patients may occur not only with administration of valproate but with administration of other AEDs (phenobarbital, phenytoin, carbamazepine) and low nutritional intake of carnitine [11].

Our results showed no statistically significant difference in carnitine level between different age groups which is not in agreement with Fung E [8], because we included only children and no adults were included in the study.

We reported carnitine deficiency in 64% of cases treated with different AEDs. All those cases were asymptomatic which agrees with Belousova E [11]. Fukuda M [12] and Coppola G [13] reported less frequency of carnitine deficiency among similar group of patients; 16.9% and 25% respectively. On the other hand, Kurul S [14] did not report the presence of carnitine deficiency among the studied groups. The recorded high percentage of carnitine deficiency in our study can be attributed to the presence of low carnitine level among cases and controls.

Our results showed statistically significant difference in carnitine level in patients receiving monotherapy compared to the controls, but no statistical difference in patients treated with polytherapy compared to controls. This result indicates that the number of AEDs used had no effect on carnitine level, and it is not a risk factor for carnitine deficiency. This is in agreement with Fung E [8] while Belousova E [11] and Fukuda M [12] showed that polytherapy is a risk factor for carnitine deficiency. We can attribute the discrepancy in results to the small number of patients receiving polytherapy and to the cumulative data of patients receiving different AEDs in our study.

We reported a statistically significant difference in carnitine level in patients receiving valproate either as mono- or polytherapy compared to the control group, and the deficiency was more apparent in the polytherapy group. These results are in agreement with Fung E [8] and Nakajima Y [15] who showed that the long-term valproate treatment could affect some specific acylcarnitines, which is enhanced by the concomitant use of other AEDs. They speculated that the formation of valproyl-carnitine alone is insufficient to develop severe carnitine deficiency at therapeutic doses of valproate. In contrast, Fukuda M [12] and Maeda Y [16] showed that the therapeutic use of valproate, either alone or in combination with other AEDs did not appear to play a role in carnitine deficiency.

Our results showed insignificant association between the level of carnitine and different doses and duration of treatment with valproate either as mono or polytherapy. This result indicates that neither the dose nor the duration of valproate treatment influences the carnitine level. This agrees with Fung E [8] but disagree with Anil M [17]. The contradictory results can be explained by the diversity of doses and regimens of different AEDs used in the studies.

Concerning the carbamazepine therapy, there was no statistical differences in carnitine level among cases treated with carbamazepine either alone or with other AEDs compared to the control group. Nevertheless, there is insignificant association between carnitine level and different doses or duration of treatment with carbamazepine. These results are concomitant with Kurul S [14] but in disagreement with Silva M [18] and Werner T [19].

Finally, there was no statistical difference in carnitine level among patients receiving other AEDs (new or old generations) compared to the control group. We speculate that these AEDs probably do not affect the carnitine level which is in agreement with Sahar E, 2012 [20] but in disagreement with Silva M [18] and Werner T [19]. The discrepancy between our results and those obtained in previous studies concerning the carbamazepine and other AEDs can be attributed to the scarce data published on this issue.

Interestingly, we detected a low level of carnitine in 40% of the controls. We suggest that this carnitine deficiency may be due to low carnitine intake as a dietary habit among children in Fayoum governate. Those children do not take a well-balanced diet containing significant amounts of carnitine as well as the essential amino acids and micronutrients needed for carnitine biosynthesis; yet, they did not reach a state of nutritional deficiency.

## Conclusions

Carnitine deficiency is present in our population and the intake of valproate with other AEDs increases its deficiency in epileptic patients. The age of the patients, the duration of treatment, and the doses of different AEDs are not risk factors for carnitine deficiency. Monitoring serum carnitine level in patients treated with different AEDs is warranted with subsequent carnitine supplementation. The limitation of our study was the small size of the sample obtained from one center study, thus, further studies are needed in multiple centers to evaluate the incidence of carnitine deficiency in the Egyptian population.

## Abbreviations

AEDs: Antiepileptic drugs
ANOVA: Analysis of variance
EEG: Encephalogram
ELISA: Enzyme-linked immune sorbent assay
HRP: Horseradish peroxidase
MRI: Magnetic resonance imaging
OD: Absorbance
RPM: Revolution per minute
TC: Total carnitine
